# A Dose-Response Meta-analysis of Coffee Consumption and Colorectal Cancer Risk in the Japanese Population: Application of a Cubic-Spline Model

**DOI:** 10.2188/jea.JE20170201

**Published:** 2018-12-05

**Authors:** Ken Horisaki, Kunihiko Takahashi, Hidemi Ito, Shigeyuki Matsui

**Affiliations:** 1 Department of Biostatistics, Nagoya University Graduate School of Medicine, Nagoya, Japan; 2 Division of Molecular and Clinical Epidemiology, Aichi Cancer Center Research Institute, Nagoya, Japan

**Keywords:** meta-analysis, dose-response, coffee consumption, colorectal cancer, cubic spline model

## Abstract

**Background:**

A recent meta-analysis compared the relative risks of colorectal cancer between the highest and lowest levels of coffee consumption in the Japanese population. However, this analysis did not define the risks with respect to specific exposure values when considering levels of coffee consumption per day in the study population.

**Methods:**

We conducted a two-stage random-effects dose-response meta-analysis of the association between coffee consumption and colorectal cancer among the Japanese. This was performed by modeling coffee consumption using restricted cubic splines to be able to examine a potential nonlinear relation.

**Results:**

We identified a total of 26 studies from seven articles, which were distributed separately according to sex and colon/rectum cancers. Data from 14 cohort studies showed that the pooled relative risks for colorectal cancers were less than 1.0 in cases with coffee consumption of 1–3 cups/day and 1.0 in cases with consumption of 4 cups/day or more, although these results were not statistically significant. Data from 12 case-control studies showed that the pooled odds ratios for cancer risk were significantly less than 1.0 in cases with coffee consumption of 1–6 cups/day.

**Conclusions:**

Findings from this meta-analysis indicate that moderate coffee consumption may not be associated or may be weakly inversely associated with the risk of colorectal cancer in the Japanese population.

## INTRODUCTION

Colorectal cancer (CRC) is one of the most commonly diagnosed malignancies and the leading cause of cancer-related deaths in the world.^1^ In Japan, the incidence and mortality rates of CRC have increased markedly over the last several decades,^2^^,^^3^ and the age-standardized incidence rate of CRC was the second-highest among all cancers in each sex.^3^ Such an increase has been attributed to environmental factors, including lifestyle and diet; moreover, strong evidence has been provided by both ecological and migrant studies.^4^^–^^7^

Coffee is one of the most frequently consumed beverages worldwide, and a number of epidemiological studies have discussed the relationship between coffee consumption and cancer risk.^8^ Recently, the International Agency for Research on Cancer (IARC) concluded that there is moderate evidence of an association between coffee drinking and a reduced risk of colorectal adenoma.^9^ Moreover, the research group for “Development and Evaluation of Cancer Prevention Strategies in Japan”^10^ evaluated cancer risks in Japanese people based on scientific evidence, and they concluded that coffee drinking was associated with a lower risk as “probable” for liver cancer and “possible” for endometrium cancer.

These conclusions are supported by a number of studies, including prospective cohort and population-based case-control studies, as well as meta-analyses. Although several meta-analyses^11^^–^^15^ have been published on the association between coffee consumption and CRC, the conclusions are inconsistent. One of the reasons for this might be due to the differences in the areas/countries studied.

Recently, a meta-analysis of epidemiological studies on coffee consumption and CRC risk conducted only for the Japanese population was reported by Akter et al.^16^ This study concluded that the findings of cohort studies were inconsistent, as they reported a relative risk (RR) of 0.95 with 95% confidence interval (CI) of 0.77–1.17, whereas findings of case-control studies suggested an inverse association, reporting an odds ratio (OR) of 0.78 with 95% CI of 0.65–0.95. Akter et al thus concluded that the evidence was insufficient to support the fact that coffee drinking either increases or decreases colorectal cancer risk among the Japanese population.

Most of the previous meta-analyses were performed using the pooled analysis of RRs or ORs as reported in each cohort and case-control study. The aforementioned meta-analysis among the Japanese population evaluated CRC risks for the highest coffee-consumption group as compared with those of the lowest. However, categories of coffee consumption per day differed between studies, and study-specific risks of the highest consumption categories relative to the lowest were adopted in the analyses. Thus, interpretation of the pooled results across study populations might have been complicated when different categories were used.^17^ Moreover, it would be of great interest to know the risks with respect to specific exposure values when considering coffee consumption per day.

A dose-response meta-analysis could provide a solution to this problem. Some recent studies^17^^,^^18^ have presented dose-response meta-analyses of worldwide populations by employing a cubic spline model to examine a potential nonlinear (including a simple linear) association between coffee consumption and CRC. However, this type of analysis had not been performed among the Japanese population. Therefore, we conducted such a dose-response meta-analysis of the association between coffee consumption and CRC in this population alone, based on the studies pooled in the recent meta-analysis by Akter et al.^16^

## METHODS

### Data

We evaluated articles of relevant epidemiological studies identified by Akter et al^16^—five cohort studies and nine case-control studies—along with the most recently published^19^ case-control study. Only studies based on Japanese residents were included. We employed articles reporting RRs or ORs along with details, such as the number of people, cases, standard errors, or CIs, in more than three categories.

### Statistical analysis

We conducted a two-stage random-effects dose-response meta-analysis^17^^,^^18^ of the association between coffee consumption and CRC (Appendix). We assessed dose-response associations using a summarized group of data that was based on the assignment of levels to grouped exposure intervals by applying the likelihood-based assignment procedure.^20^^,^^21^ We employed the restricted cubic spline model for potential nonlinear dose-response curves for selecting locations of the three knots appropriately in order to maximize the likelihood.^21^^,^^22^ This procedure can estimate the log-RR with respect to the exposure level of zero for each study, even when the level of reference category for reported RRs or ORs is not zero. Statistical heterogeneity among studies was assessed using the multivariate Cochran’s Q-test. Additionally, goodness of fit assessments were conducted by indicating as deviance.^23^ These analyses were performed using the package “mvmeta”^24^ in software R, version 3.3.1 (R Foundation for Statistical Computing, Vienna, Austria).^25^

We performed dose-response meta-analyses for cohort studies and case-control studies separately. For sub-group analyses, we performed combined analyses among studies of colon and rectum cancers, respectively. In addition, we performed separate analyses for each gender in the cohort studies. However, we were unable to perform these analyses for case-control studies, because only one study reported separate results by gender.

## RESULTS

We identified a total of 26 studies from seven articles, which extracted 14 cohort-based and 12 case-control studies by sex and anatomical sites (colon/rectum cancers) from four cohort articles and three case-control articles,^19^^,^^26^^–^^31^ separately. Among the cohort studies, three articles^27^^–^^29^ separated results of studies for cancers of the colon and rectum, and one article^26^ reported only on colon cancer. All of these articles reported results of studies for men and women separately. A total of 14 cohort studies were included in this analysis (Table 1). Among the case-control studies, three articles^19^^,^^30^^,^^31^ reported results of studies for colon and rectum cancers separately. Two articles^30^^,^^31^ presented combined results for men and women. One article^19^ reported results for two study populations, HERPACC-I and HERPACC-II, separately. A total of 12 case-control studies were employed in our meta-analysis (Table 2). Although the adjusted factors for reported risks in each study were different, all studies commonly adjusted for age, smoking, and alcohol intake.

**Table 1.** Characteristics of cohort studies evaluating coffee consumption and colorectal cancer risk included in a meta-analysis

	Study period	Sex	Coffee intake	RR (95% CI)	Reference category
**Colon**					
Oba et al^26^	1992–2000	Men	1 cup/month–1 cup/day	1.13 (0.71, 1.81)	<1 cup/month
			>1 cup/day	0.81 (0.46, 1.42)	
		Women	1 cup/month–1 cup/day	0.92 (0.59, 1.43)	<1 cup/month
			>1 cup/day	0.43 (0.22, 0.85)	
Naganuma et al^27^	1990–2001	Men	Occasionally	1.20 (0.78, 1.85)	Never
			1–2 cups/day	1.06 (0.66, 1.72)	
			>3 cups/day	0.91 (0.49, 1.69)	
		Women	Occasionally	1.40 (0.83, 2.36)	Never
			1–2 cups/day	1.05 (0.56, 1.95)	
			>3 cups/day	1.16 (0.47, 2.88)	
Lee et al^28^	1990–2002	Men	<1 cup/day	1.12 (0.87, 1.42)	Almost never
			1–2 cups/day	0.94 (0.70, 1.25)	
			>3 cups/day	1.15 (0.80, 1.66)	
		Women	<1 cup/day	0.87 (0.64, 1.19)	Almost never
			1–2 cups/day	0.94 (0.67, 1.33)	
			>3 cups/day	0.60 (0.31, 1.19)	
Yamada et al^29^	1988–2009	Men	1 cup/day	1.06 (0.76, 1.47)	<1 cup/day
			2–3 cups/day	1.26 (0.93, 1.70)	
			>4 cups/day	1.79 (1.01, 3.18)	
		Women	1 cup/day	1.00 (0.72, 1.37)	<1 cup/day
			2–3 cups/day	0.86 (0.57, 1.30)	
			>4 cups/day	2.02 (0.81, 5.03)	

**Rectum**					
Naganuma et al^27^	1990–2001	Men	Occasionally	1.07 (0.61, 1.87)	Never
			1–2 cups/day	0.93 (0.51, 1.71)	
			>3 cups/day	0.92 (0.45, 1.90)	
		Women	Occasionally	0.84 (0.44, 1.60)	Never
			1–2 cups/day	0.85 (0.42, 1.72)	
			>3 cups/day	1.08 (0.42, 2.82)	
Lee et al^28^	1990–2002	Men	<1 cup/day	0.69 (0.48, 0.99)	Almost never
			1–2 cups/day	0.94 (0.64, 1.36)	
			>3 cups/day	1.01 (0.61, 1.66)	
		Women	<1 cup/day	1.04 (0.67, 1.62)	Almost never
			1–2 cups/day	1.16 (0.72, 1.88)	
			>3 cups/day	0.84 (0.36, 1.94)	
Yamada et al^29^	1988–2009	Men	1 cup/day	1.19 (0.79, 1.80)	<1 cup/day
			2–3 cups/day	1.12 (0.75, 1.70)	
			>4 cups/day	1.19 (0.48, 2.95)	
		Women	1 cup/day	0.88 (0.48, 1.59)	<1 cup/day
			2–3 cups/day	1.55 (0.89, 2.69)	

CI, confidence interval; RR, relative risk.

**Table 2.** Characteristics of case-control studies evaluating coffee consumption and colorectal cancer risk included in a meta-analysis

	Study period	Sex	Coffee intake	OR (95% CI)	Reference category
**Colon**					
Inoue et al^30^	1990–1995	Men and Women	Occasionally	0.83 (0.61, 1.14)	Rarely
			1–2 cups/day	0.97 (0.74, 1.27)	
			>3 cups/day	0.87 (0.56, 1.35)	
Wang et al^31^	2000–2003	Men and Women	1–3 cups/week	1.04 (0.69, 1.57)	<1 cup/week
			4–6 cups/week	0.75 (0.47, 1.18)	
			1–3 cups/day	0.64 (0.47, 0.87)	
			>4 cups/day	0.78 (0.53, 1.35)	
Nakagawa et al^19^ HERPACC-I	1988–2000	Men	<1 cup/day	0.78 (0.55, 1.10)	None
			1–2 cups/day	0.82 (0.62, 1.09)	
			>3 cups/day	0.97 (0.64, 1.46)	
		Women	<1 cup/day	0.96 (0.68, 1.37)	None
			1–2 cups/day	0.91 (0.67, 1.25)	
			>3 cups/day	0.63 (0.35, 1.13)	
Nakagawa et al^19^ HERPACC-II	2001–2005	Men	<1 cup/day	0.60 (0.37, 0.97)	None
			1–2 cups/day	0.62 (0.38, 0.99)	
			>3 cups/day	0.59 (0.33, 1.06)	
		Women	<1 cup/day	1.18 (0.68, 2.04)	None
			1–2 cups/day	1.28 (0.76, 2.17)	
			>3 cups/day	1.22 (0.59, 2.51)	

**Rectum**					
Inoue et al^30^	1990–1995	Men and Women	Occasionally	0.77 (0.53, 1.11)	Rarely
			1–2 cups/day	0.91 (0.67, 1.24)	
			>3 cups/day	0.46 (0.26, 0.81)	
Wang et al^31^	2000–2003	Men and Women	1–3 cups/week	0.63 (0.38, 1.04)	<1 cup/week
			4–6 cups/week	0.55 (0.32, 0.93)	
			1–3 cups/day	0.63 (0.45, 0.88)	
			>4 cups/day	0.82 (0.54, 1.23)	
Nakagawa et al^19^ HERPACC-I	1988–2000	Men	<1 cup/day	0.84 (0.60, 1.17)	None
			1–2 cups/day	0.91 (0.69, 1.19)	
			>3 cups/day	0.60 (0.41, 0.89)	
		Women	<1 cup/day	1.18 (0.80, 1.74)	None
			1–2 cups/day	0.91 (0.64, 1.28)	
			>3 cups/day	0.88 (0.48, 1.60)	
Nakagawa et al^19^ HERPACC-II	2001–2005	Men	<1 cup/day	0.91 (0.55, 1.51)	None
			1–2 cups/day	1.06 (0.65, 1.73)	
			>3 cups/day	0.98 (0.54, 1.78)	
		Women	<1 cup/day	1.32 (0.69, 2.50)	None
			1–2 cups/day	0.94 (0.49, 1.24)	
			>3 cups/day	0.91 (0.42, 2.02)	

CI, confidence interval; OR, odds ratio.

**Figure 1.**
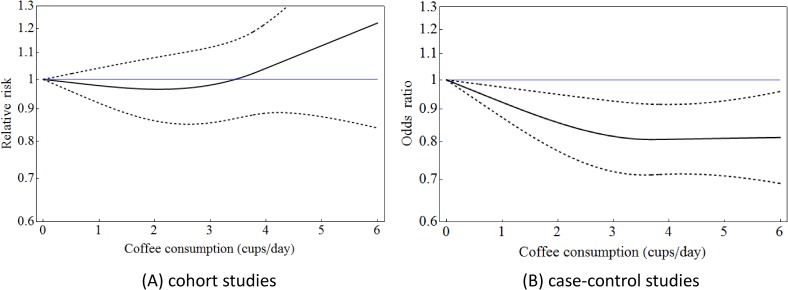
Dose-response curves of colorectal cancer risk associated with coffee consumption among the Japanese population, in a meta-analysis including cohort studies (A) and case-control studies (B). Dashed curves represent 95% confidence intervals for each value of coffee consumption.

For the overall association between coffee consumption and total CRC (ie, cancers of the colon and rectum), we were able to estimate a pooled dose-response curve from the 14 cohort studies using a cubic spline regression method with coefficients (β_1_, β_2_) = (−0.023, 0.003) and three knots (*k*_1_, *k*_2_, *k*_3_) = (0.51, 3.77, 3.94) (Figure 1A). *P*-value of the multivariate Cochran’s Q-test for heterogeneity was *P_Q_* = 0.639. The cubic spline model was a better fit, as indicated by a deviance of *D* = 32.98 (*P* = 0.658), than the simple linear model (*D* = 45.74, *P* = 0.182). The pooled RRs of total CRC were less than 1.0 for coffee drinkers of 1–3 cups/day and >1.0 for 4 cups/day or more consumed, as compared to subjects with no coffee consumption; however, these results were not significant, with α = 0.05 for all levels of coffee consumption.

On the other hand, a pooled curve from the 12 case-control studies had coefficients (β_1_, β_2_) = (−0.081, 0.003) with (*k*_1_, *k*_2_, *k*_3_) = (0.59, 3.68, 3.83) (Figure 1B). *P* for heterogeneity was *P_Q_* = 0.162. The deviance of *D* = 43.34 (*P* = 0.187) for the cubic spline model showed a better fit than the simple linear model (*D* = 50.86, *P* = 0.064). The pooled ORs of total CRC were significantly less than 1.0 for coffee drinkers of 1–6 cups/day as compared to subjects with no coffee consumption.

**Figure 2.**
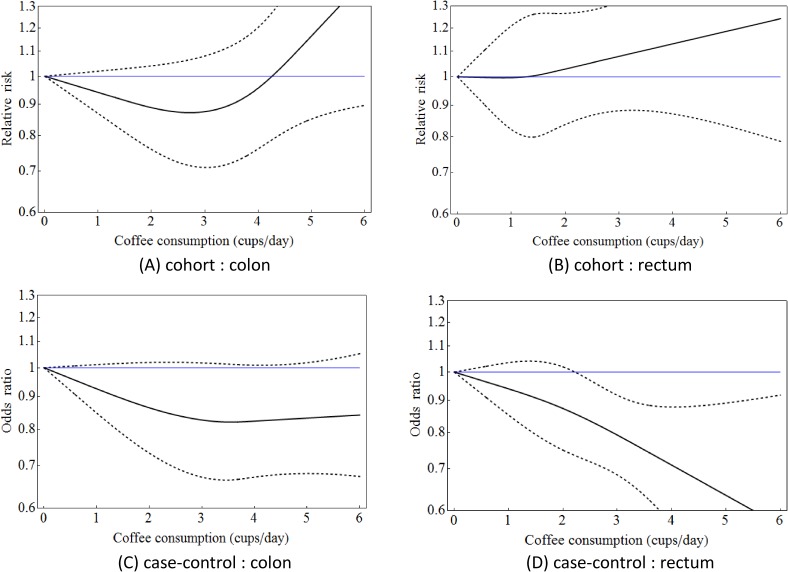
Dose-response curves of colorectal cancer risks associated with coffee consumption in the Japanese population for colon (A) and rectum (B) (meta-analysis based on cohort studies), and for colon (C) and rectum (D) (meta-analysis based on case-control studies). Dashed curves represent 95% confidence intervals for each value of coffee consumption.

Figure 2 shows the results of subgroup analyses. Although cohort studies did not reveal any significant associations between coffee consumption and cancers of either the colon or the rectum, the risk of colon cancer tended to decrease with the consumption of 1–4 cups of coffee per day. The risk of rectum cancer, however, tended to increase with coffee consumption. In case-control studies, risks of both colon and rectum cancers decreased with coffee consumption. In particular, coffee consumption of 3 cups/day or more decreased the risk of rectum cancer significantly. Moreover, there were no significant differences found in heterogeneity between the two cancers; *P_Q_* = 0.351 (colon) and 0.843 (rectum) in cohort studies, while these values in case-control studies were 0.155 (colon) and 0.117 (rectum).

Table 3 shows RRs or ORs associated with consumption of 1–6 cups of coffee per day. Associated risks for 1–3 cups/day were similar across the study design, except for the risk of rectum cancer, as reported in cohort studies. However, results at high levels of coffee consumption were inconclusive because of the few available data points in the higher-consumption categories, as shown in Table 1.

**Table 3.** Relative risks or odds ratios of colorectal cancer associated with consumption of 1–6 cups of coffee per day

	Number of Studies	Coffee Consumption, cups/day											

		1		2		3		4		5		6	
					
		RR/OR	95% CI	RR/OR	95% CI	RR/OR	95% CI	RR/OR	95% CI	RR/OR	95% CI	RR/OR	95% CI
**Cohort**													
All studies	14	0.98	0.92, 1.04	0.97	0.96, 1.08	0.98	0.86, 1.12	1.04	0.89, 1.22	1.13	0.87, 1.46	1.22	0.84, 1.78

Colon	8	0.94	0.87, 1.02	0.89	0.76, 1.04	0.88	0.71, 1.08	0.96	0.76, 1.20	1.16	0.85, 1.59	1.44	0.90, 2.31
Rectum	6	1.00	0.82, 1.21	1.03	0.84, 1.27	1.08	0.88, 1.32	1.13	0.87, 1.47	1.18	0.83, 1.68	1.24	0.79, 1.96

Men	7	1.01	0.91, 1.12	1.02	0.87, 1.20	1.04	0.89, 1.22	1.07	0.87, 1.30	1.09	0.84, 1.43	1.12	0.79, 1.58
Women	7	0.95	0.85, 1.06	0.90	0.73, 1.12	0.89	0.70, 1.14	0.97	0.67, 1.40	1.09	0.49, 2.42	1.22	0.35, 4.33

**Case-control**													
All studies	12	0.92	0.87, 0.97	0.86	0.77, 0.95	0.82	0.72, 0.93	0.81	0.71, 0.92	0.81	0.71, 0.93	0.81	0.69, 0.96

Colon	6	0.93	0.85, 1.01	0.86	0.73, 1.02	0.83	0.67, 1.02	0.82	0.67, 1.01	0.83	0.68, 1.02	0.84	0.67, 1.05
Rectum	6	0.94	0.85, 1.04	0.87	0.75, 1.02	0.79	0.68, 0.92	0.71	0.57, 0.88	0.63	0.45, 0.89	0.57	0.35, 0.92

CI, confidence interval; OR, odds ratio; RR, relative risk.

## DISCUSSION

In this study, a meta-analysis of cohort studies indicated that associations between coffee consumption and CRC were not significant, whereas meta-analysis of case-control studies suggested an inverse association in the Japanese population. These findings coincide with those of the report by Akter et al,^16^ which only compared RR or OR between the highest consumption group and the lowest. Our dose-response meta-analysis indicated the CRC risk by specific levels of consumption, and results suggested that mild coffee consumption may not be associated or may be weakly inversely associated with risk of CRC.

Case-control studies, in particular those employing summarized ORs, have reported a statistically significant decrease in risk of CRC with levels of coffee consumption of 1–6 cups/day. Moreover, they showed an inverse association with both colon and rectum cancers. As pointed out by Akter et al,^16^ this finding is generally consistent with previous meta-analyses of case-control studies,^9^^,^^14^^,^^15^ including Japanese studies. In contrast to Akter’s analysis, we employed studies from three articles, including a new report, based on which we excluded five articles. Although this is a limitation of our analysis, it should be noted that all articles excluded were older reports, published from 1984 through 1997, and included articles that were published after 1998. This suggests that the same risk reduction might also be found in a recent study of the Japanese population. Therefore, due to limitations inherent in case-control studies, findings from these studies should be interpreted with caution. Moreover, differences in preferences of coffee consumption between populations where cases and controls were selected could raise the possibility of selection bias.

In cohort studies, on the other hand, we should be cautious because we cannot be sure that coffee-drinking habits did not change after baseline. Moreover, we should be deeply concerned about whether it was common to drink coffee in the early 1990s in Japan, as well as what the characteristics of the population with coffee habits were at that time. The cohort-study populations were generally older in age than those of the case-control studies, even if, for example, both studies enrolled 40-year-olds, because the age of the entry was recorded in the cohort study. Considering this older population, particularly in men, prevalence of tobacco consumption and/or exposure might be high, thus presenting a confounding effect that could not be sufficiently adjusted for. Moreover, as compared to the colon, the rectum is the most affected by smoking, and, in the cohort studies, only cancer of the rectum showed an increased association between coffee consumption and CRC.

As a strength of this study, our dose-response meta-analysis reveals the CRC risk in specific levels of coffee consumption, rather than the relative risks between the highest and lowest levels of consumption provided in traditional meta-analysis. Our meta-analysis assessed potential nonlinear associations between coffee consumption and the risk of CRC, employing the restricted cubic spline model. On the other hand, as a limitation of this study, the results of a meta-analysis must be sensitive to the model, including the number of knots, although some studies have applied similar models to evaluate the dose-response of coffee consumption and disease.^22^^,^^32^^,^^33^ Therefore, further discussion of these models, including evaluation of different methods, is warranted.

### Conclusion

From the results of the present dose-response meta-analysis, we conclude that there is insufficient evidence to support the hypothesis that coffee drinking either increases or decreases CRC risk among the Japanese population. Moreover, our results suggest that mild coffee consumption may not be associated with or only weakly inversely associated with the risk of CRC, although its influence is not statistically significant.

## APPENDIX. PROCEDURE FOR DOSE-RESPONSE META-ANALYSIS

For dose-response analysis, we used the restricted cubic spline model for potential nonlinear regression modeling. First, we assigned exposure levels in each study, because all the studies reported only summarized data. The data on exposure levels of coffee consumption were available as categories by interval. When performing a regression analysis of summarized response data that are grouped into intervals, many researchers frequently use the pre-assigned exposure levels from the midpoint values of each interval. Because results of this type of analysis may be sensitive to the assignment of values to grouped intervals of exposure, we employed likelihood based assignment of levels for grouped exposure intervals,^20^^,^^21^ which can estimate upper-ended categories of exposure variables together.

In a manner similar to that of previous dose-response meta-analyses,^17^^,^^18^ we performed a two-stage random-effects dose-response meta-analysis considering between-study heterogeneity. We modeled log-relative risk against coffee consumption using restricted cubic splines with three knots. In previous studies,^17^^,^^18^^,^^22^ knots were placed at fixed percentiles in the data. We employed a procedure for selecting the positions of three knots among the following combinations: (10%, 20%, 30%), (10%, 20%, 40%)…(70%, 80%, 90%) of the overall distribution of assigned exposure levels for studies included in our meta-analysis. The common knots across studies, *k*_1_, *k*_2_ and *k*_3_, were placed in a position that maximizes the total of the log-likelihood function, $\sum_{i}l({\hat{b}}_{i}|k_{1},k_{2},k_{3})$, where ${\hat{b}}_{i}$ is an estimate of regression coefficients for *i*-th study (see details in Takahashi et al^21^). Note that this procedure can be used to estimate a spline model for the log-relative risk with respect to the exposure level of zero for each study, even when the level of reference category for reported RRs or ORs is not zero.

Next, we combined the two regression coefficients and the variance/covariance matrix that had been estimated within each study by using the restricted maximum likelihood method in a multivariate random-effects meta-analysis.^34^^,^^35^
